# FOXC2 regulates the G2/M transition of stem cell-rich breast cancer cells and sensitizes them to PLK1 inhibition

**DOI:** 10.1038/srep23070

**Published:** 2016-04-11

**Authors:** Mika Pietilä, Geraldine V. Vijay, Rama Soundararajan, Xian Yu, William F. Symmans, Nathalie Sphyris, Sendurai A. Mani

**Affiliations:** 1 Department of Translational Molecular Pathology, The University of Texas MD Anderson Cancer Centre, Houston, TX, USA; 2 Metastasis Research Centre, The University of Texas MD Anderson Cancer Centre, Houston, TX, USA; 3 Center for Stem Cells and Developmental Biology, The University of Texas MD Anderson Cancer Centre, Houston, TX, USA

## Abstract

Cancer cells with stem cell properties (CSCs) underpin the chemotherapy resistance and high therapeutic failure of triple-negative breast cancers (TNBCs). Even though CSCs are known to proliferate more slowly, they are sensitive to inhibitors of G2/M kinases such as polo-like kinase 1 (PLK1). Understanding the cell cycle regulatory mechanisms of CSCs will help target these cells more efficiently. Herein, we identify a novel role for the transcription factor FOXC2, which is mostly expressed in CSCs, in the regulation of cell cycle of CSC-enriched breast cancer cells. We demonstrate that FOXC2 expression is regulated in a cell cycle-dependent manner, with FOXC2 protein levels accumulating in G2, and rapidly decreasing during mitosis. Knockdown of FOXC2 in CSC-enriched TNBC cells delays mitotic entry without significantly affecting the overall proliferation rate of these cells. Moreover, PLK1 activity is important for FOXC2 protein stability, since PLK1 inhibition reduces FOXC2 protein levels. Indeed, FOXC2 expressing CSC-enriched TNBC cells are sensitive to PLK1 inhibition. Collectively, our findings demonstrate a novel role for FOXC2 as a regulator of the G2/M transition and elucidate the reason for the observed sensitivity of CSC-enriched breast cancer cells to PLK1 inhibitor.

Cancer cells with stem cell properties (CSCs) are a subpopulation of tumour cells that sustain long-term tumour propagation through their ability to self-renew and to generate differentiated progeny that comprise the bulk of the tumour^1^,^2^. Importantly, CSCs are intrinsically resistant to cytotoxic therapies and the residual tumours, remaining after treatment with conventional therapies, are enriched for CSCs^3^,^4^,^5^,^6^. Accordingly, several studies have found that CSC gene expression signatures serve as independent predictors of poor disease-free or overall patient survival in multiple tumour types^7^,^8^,^9^,^10^,^11^,^12^.

CSCs are regarded as slow-cycling cells that can transiently and reversibly enter a quiescent or dormant state, thought to underpin their resistance to cytotoxic therapies and tumour recurrence^6^,^13^,^14^,^15^. Paradoxically, recent studies have shown that CSCs are sensitive to inhibition of polo-like kinase 1 (PLK1)—a key regulator of the G2/M transition^16^,^17^—whereas they are highly resistant to traditional anti-mitotic drugs such as the microtubule-stabilizing agent paclitaxel^4^,^18^. Indeed, gene expression profiling studies and screens employing small molecule kinase inhibitors or small interfering RNA (siRNA) libraries have demonstrated that PLK1 inhibition may lead to the elimination of CSCs in a range of tumours, including neuroblastoma^19^, glioblastoma^20^, as well as breast cancer^21^,^22^.

While the above findings support the notion that inhibition of mitotic kinases may target CSCs, they also pose an apparent paradox with the widely purported view that CSCs reside in a quiescent/dormant state^13^,^14^,^15^. A recent study appears to reconcile this paradox by demonstrating that chemotherapy stimulates CSC proliferation—similar to the activation of normal stem cells following tissue damage—leading to tumour recurrence and CSC enrichment^23^. While many chemotherapeutic agents specifically target cycling cells, it is unclear whether CSCs exhibit a unique cell cycle profile and how are they susceptible to G2/M-specific kinase inhibitors; these warrants an improved understanding of the factors governing cell cycle progression in CSCs.

We and others have shown that the aberrant activation of a latent embryonic program—known as the epithelial-mesenchymal transition (EMT)—confers migratory and invasive capabilities^24^ as well as stem cell/tumour-initiating properties upon differentiated tumour cells^25^,^26^. We recently identified the transcription factor Forkhead Box C2 (FOXC2) as a key regulator of EMT and stem cell properties, including tumour-initiation capacity, metastatic competence, and chemotherapy resistance^27^,^28^. Most importantly, FOXC2 levels were found to be elevated in TNBCs^27^,^28^, as well as in residual tumour cells isolated from breast cancer patients treated with conventional therapies, which were found to be enriched for mesenchymal and stem cell features^12^. These findings implicate FOXC2 in therapy resistance, tumour recurrence and TNBC progression.

Although the role of FOXC2 in the regulation of EMT and CSC properties is well established, little is known about the processes governing FOXC2 regulation in CSCs. Recently, it was shown that only wild-type FOXC2, but not a phosphorylation-deficient mutant, was able to induce the expression of several cell cycle regulators, including cyclin-dependent kinase 1 (CDK1), suggesting a role for FOXC2 phosphorylation in the expression of cell cycle-specific genes^29^. Moreover, FOXC2 expression has been shown to enhance proliferation in several types of tumour cells^30^,^31^,^32^,^33^,^34^,^35^. Collectively, these studies indirectly link FOXC2 to the regulation of cell cycle and proliferation, although its role in the cell cycle of CSCs remains unclear. In this work, we investigated the relationship between FOXC2 expression and the cell cycle of CSC-enriched TNBC cells and cells that have undergone EMT. We found that FOXC2 not only regulates the G2/M transition in these cells, but also its expression is post-translationally regulated in a cell cycle-dependent manner, at least in part, by PLK1. We also show that FOXC2 expression sensitizes CSC-enriched TNBC cells to the PLK1 inhibitor BI 2536.

## Results

### Cell cycle-dependent fluctuation of FOXC2 protein levels in stem cell-enriched cell lines

**Figure 1:**
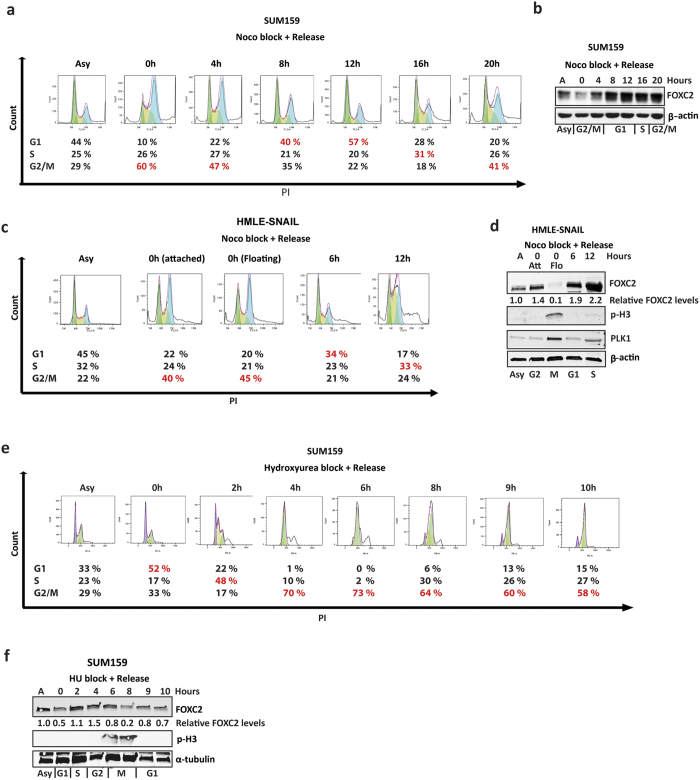
FOXC2 protein levels fluctuate in a cell cycle-dependent manner, accumulating at G2 and rapidly decreasing when cells enter mitosis. (**a**) SUM159 cells were synchronised by nocodazole at G2/M and then released into normal medium. The cells were harvested at various timepoints after nocodazole-release and subjected to PI staining. The cellular DNA content was analysed by flow cytometry to quantify the number of cells in the respective phases of the cell cycle. Cell-cycle distributions are summarized at the bottom. Asy, asynchronised cells. (**b**) SUM159 cells were harvested at different timepoints after nocodazole-arrest and –release, and the corresponding lysates were analysed by immunoblotting for FOXC2. β-actin was used as a loading control. The cell cycle stage is indicated below the blot. A, asynchronised cells. (**c**) HMLE-SNAIL cells were synchronised with nocodazole and then released into normal medium. Immediately after nocodazole treatment, the attached or floating cells were harvested. In addition, cells were harvested 6 and 12 h post-nocodazole release. Cells were subjected to PI staining and cell cycle analysis by flow cytometry. Cell-cycle distributions are summarized at the bottom. Asy, asynchronised cells. (**d**) Cell lysates from nocodazole-treated HMLE-SNAIL cells were analysed by immunoblotting for FOXC2, PLK1 and p-H3, a marker of mitotic cells. β-actin was used as a loading control. The cell cycle stage, at which cells were arrested by nocodazole, is indicated below the blot. Number below FOXC2 blot indicates the level of FOXC2 normalized to actin relative to asynchronous cells. (**e**) SUM159 cells were arrested in G1/S by hydroxyurea, released into normal medium, and harvested at various timepoints post-hydroxyurea release. Cells were subjected to PI staining and cell cycle analysis by flow cytometry and (**f**) cell lysates were analysed by immunoblotting for FOXC2 and p-H3. a-tubulin was used as a loading control. The cell cycle stage, at which cells were arrested by hydroxyurea is indicated below the blot.

To investigate whether FOXC2 protein levels are regulated during the cell cycle, we synchronised FOXC2-expressing CSC-rich human TNBC SUM159 cells using nocodazole, which interferes with microtubule polymerization, thus arresting cells in G2/M phase. To enrich for cells in G2/M, as well as the other stages of the cell cycle, we exposed cells to nocodazole and confirmed that 60% of the cells are in G2/M phase by FACS analysis (Fig. 1a). The cells were released into normal growth medium to allow their progression through the cell cycle. Cells were harvested at different time points, as indicated (Fig. 1a), and the levels of FOXC2 examined by immunoblotting. Indeed, nocodazole-induced G2/M arrest led to a dramatic decrease in FOXC2 protein levels in SUM159 cells (Fig. 1b). Following release from nocodazole block, FOXC2 protein levels gradually increased and peaked at G2 (Fig. 1b). The cell cycle-dependent fluctuation of FOXC2 protein levels was also evident in immortalized human mammary epithelial (HMLE) cells overexpressing the EMT-inducing transcription factor SNAIL (HMLE-SNAIL). In this model, we found that nocodazole treatment led to an enrichment of cells in late G2 (attached) and prometaphase/mitotic cells (rounded up/floating). In addition, we enriched HMLE-SNAIL cells in G1 and S by harvesting them, 6 or 12 h after nocodazole block, respectively (Fig. 1c). Cell cycle analysis, together with immunoblotting for cell cycle markers, confirmed that the attached nocodazole-arrested cells were primarily in the G2/M phase, but did not express phospho-histone H3 (p-H3), indicating that these cells were in late G2 (Fig. 1c,d). On the other hand, floating nocodazole-arrested cells showed enrichment of a G2/M population, accompanied by high p-H3 expression, indicating that these cells were arrested at mitosis (Fig. 1c,d). Interestingly, FOXC2 was abundant in late G2/attached cells but was almost undetectable in floating mitotic cells (Fig. 1d). Similar to SUM159 cells, FOXC2 protein levels increased after release into G1 and S (Fig. 1c,d). To further study the cell cycle-dependent regulation of FOXC2 protein, and monitor more closely the changes in FOXC2 levels during the S/G2/M phases, we synchronised SUM159 cells in late G1/S by hydroxyurea treatment and subsequently released the cells. Cell cycle analysis showed that after hydroxyurea treatment, cells were arrested at the late G1 and after release cells were accumulated first in S phase and later in G2 phase (Fig. 1e). Immunoblotting analysis confirmed that FOXC2 protein levels gradually increased during G2 before its levels decreased as cells entered mitosis, with p-H3 levels serving as an indicator of mitosis (Fig. 1f). These results show that FOXC2 protein levels are regulated in a cell cycle-dependent manner in stem cell-rich cell lines.

### FOXC2 protein disappears during early mitosis and reappears at late mitosis

**Figure 2:**
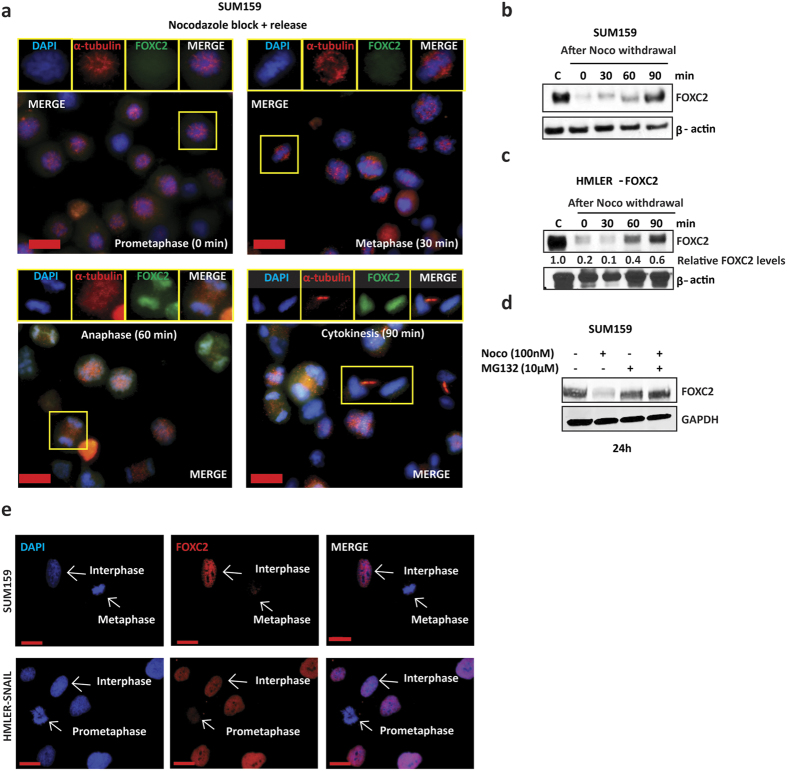
FOXC2 is degraded when cells enter mitosis and stabilized again after anaphase, co-localizing with condensed chromatin. (**a**) Immunofluorescence staining for FOXC2 (green) and alpha-tubulin (red) in nocodazole-arrested and -released SUM159 cells, counterstained with DAPI (blue). Mitotic cells were harvested by mitotic shake-off and then released. Immediately after mitotic shake-off, cells were mostly in pro-metaphase. Thirty minutes after nocodazole-release, cells were predominantly in metaphase, and after 60 min in anaphase/telophase. After 90 min, cells were in cytokinesis. FOXC2 immunostaining was weak/absent at 0 and 30 min post-nocodazole release but intensified after 60 min showing a co-localization with condensed chromatin. Representative cells of different mitotic stages are highlighted in yellow-lined insertions. Scale bar, 10 μm. (**b**,**c**) Immunoblotting for FOXC2, after mitotic shake-off and nocodazole-release, confirms the stabilization of FOXC2 protein levels 60–90 min post-nocodazole release in SUM159 cells (**b**) and in HMLER-FOXC2 cells (**c**). β-actin was used as a loading control. Number below FOXC2 blot indicates the level of FOXC2 normalized to actin relative to asynchronous cells. (**d**) SUM159 cells were treated either with nocodazole alone (100 nM), proteasome inhibitor MG132 (10 μM) alone or with nocodazole and the proteasome inhibitor MG132 for 24 h. Cells were harvested and the respective lysates analysed by immunoblotting for FOXC2. GAPDH was used as a loading control. (**e**) Immunofluorescence staining for FOXC2 (red) in asynchronised SUM159 and HMLER-SNAIL cells showing low intensity of FOXC2 immunostaining in pro-metaphase or metaphase cells (indicated with white arrows). Nuclei were counterstained with DAPI (blue). Scale bar, 10 μm.

To closely monitor changes in FOXC2 protein levels during mitosis, we arrested SUM159 cells with nocodazole and collected the floating mitotic cells. We then fixed the cells 0, 30, 60 and 90 minutes after nocodazole release to enrich for cells in the different phases of mitosis. By co-staining for FOXC2 and alpha-tubulin (Fig. 2a), we found that FOXC2 was hardly detectable during prometaphase (0 min) followed by a progressive increase in FOXC2 staining intensity as cells transitioned through metaphase (30 min), anaphase/telophase (60 min) and cytokinesis (90 min). Interestingly, we observed FOXC2 staining associated with condensed chromatin after anaphase, which is typical of transcription factors involved in mitotic bookmarking^36^ (Fig. 2a).

A similar trend was observed by immunoblotting for FOXC2 in SUM159 cells (Fig. 2b), and in Ras-transformed human mammary epithelial (HMLER) cells induced to undergo EMT by the ectopic expression of FOXC2 (HMLER-FOXC2; Fig. 2c), harvested at the same time points following nocodazole block and release, corresponding to the different phases of mitosis. These data show that FOXC2 levels rapidly decrease upon mitotic entry but begin to accumulate again, as cells transition through mitosis, and remain elevated until the late stages of mitosis. Therefore, we hypothesized that FOXC2 might be actively degraded during early mitosis. To test this, we treated SUM159 cells with either nocodazole alone, proteasome inhibitor MG132 alone or in combination, to see if FOXC2 degradation could be prevented. Indeed, we found that nocodazole-treated cells exhibited a marked decrease in FOXC2 levels, as expected, but that addition of MG132 prevented the reduction in FOXC2 protein levels (Fig. 2d). To ensure that the loss of FOXC2 was not an artifact of nocodazole exposure, we performed immunofluorescent staining for FOXC2 in asynchronised SUM159 and HMLER-SNAIL cells and found that FOXC2 was barely detectable in cells during prometaphase and metaphase, whereas interphase cells exhibited clearly discernible staining for FOXC2 (Fig. 2e). These findings show that FOXC2 undergoes rapid proteasomal degradation, when cells enter mitosis, but is stabilized after anaphase.

### PLK1 activity is needed for maintaining FOXC2 protein stability

The fact that FOXC2 protein levels are altered during the cell cycle—with no change in *FOXC2* mRNA (data not shown) and can be restored with proteome inhibitors—suggests that FOXC2 may be regulated post-translationally in a cell cycle-dependent manner. To investigate this, we analysed the FOXC2 aminoacid sequence for the presence of G2/M-specific kinase sites using SCANSITE 3 (MIT)^37^ and identified putative phosphorylation sites for PLK1, CDK1 and Aurora A (Supplementary Fig. 1a). Next, we examined the evolutionary conservation of these putative phosphorylation sites, using BLAST alignment of UniProt FOXC2 aminoacid sequences from different species. We found that only the putative PLK1 phosphorylation site at serine 125 is evolutionarily well conserved (Supplementary Fig. 1b)—suggesting PLK1 may be functionally relevant to FOXC2 function—whereas the CDK1 and Aurora A sites may not (Supplementary Fig. 1c).

**Figure 3:**
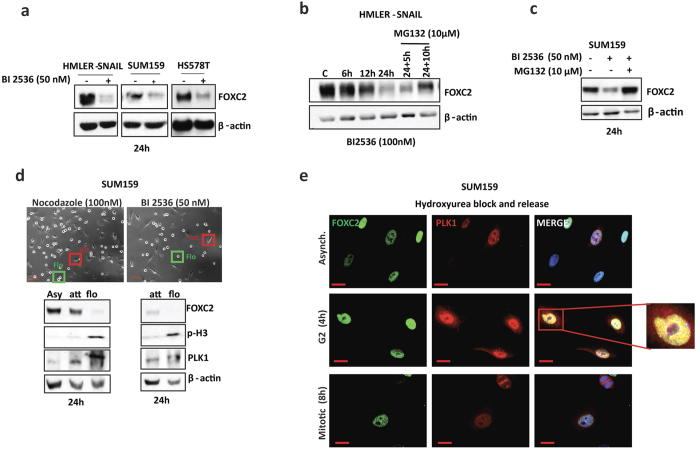
Inhibition of PLK1 causes a decrease in FOXC2 protein levels. (**a**) Treatment of HMLER-SNAIL, SUM159 and Hs578T cells with the PLK1 inhibitor BI 2536 (50 nM) for 24 h causes a dramatic decrease in FOXC2 protein levels, as detected by immunoblotting. β-actin was used as a loading control. (**b**) HMLER-SNAIL cells were treated with vehicle or BI 2536 (100 nM) for 6 h, 12 h and 24 h. After 24 h of BI 2536 treatment, the proteasome inhibitor MG132 (10 μM) was added to the BI 2536-treated cells. Immunoblotting showed that the FOXC2 protein levels of BI 2536-treated cells were increased after addition of MG132. β-actin was used as a loading control. (**c**) Immunoblotting for FOXC2 in SUM159 cells treated with either BI 2536 (50 nM) alone or with BI 2536 and MG132 (10 μM) for 24 h. β-actin was used as a loading control. (**d**) SUM159 cells were treated with either nocodazole (100 nM) or BI 2536 (50 nM). In both cases, attached (late G2) and floating (mitotic) cells were collected. The cell lysates were analysed by immunoblotting for p-H3 (mitosis), PLK1 (G2/M) and FOXC2. β-actin was used as a loading control. Asy, asynchronised cells; att, attached; flo, floating. Scale bar 100 μm. (**e**) Immunofluorescence staining for FOXC2 (green) and PLK1 (red) in asynchronised populations as well as in hydroxyurea-arrested and -released SUM159 cells. Nuclei were counterstained with DAPI (blue). The co-localization of FOXC2 and PLK1, in the nuclei of SUM159 cells 4 h after hydroxyurea release, is highlighted in the magnified insert. Scale bar, 10 μm.

To test, whether inhibition of PLK1 could affect the FOXC2 protein stability, we treated cells expressing endogenous FOXC2 such as HMLER-SNAIL, SUM159 and Hs578T with the PLK1 inhibitor BI 2536 (50 nM) for 24 h and observed a dramatic decrease in FOXC2 protein levels (Fig. 3a), suggesting that PLK1 activity is important for FOXC2 protein stability. On the other hand, CDK1 inhibitor treatment did not have a significant effect on FOXC2 protein stability (Supplementary Fig. 1d). Also, when HMLER-SNAIL cells were first treated with BI 2536 (100 nM) for 24 h, and subsequently treated for 5 or 10 h with the proteasome inhibitor MG132 (10 μM), FOXC2 protein levels were allowed to accumulate, consistent with the fact that inhibition of PLK1 activity by BI 2536 induces proteasomal degradation of FOXC2 (Fig. 3b). Similarly, FOXC2 protein levels decreased in SUM159 cells treated with BI 2536 (50 nM) alone for 24 h, whereas co-treatment of these cells with BI 2536 and MG132 (10 μM) prevented FOXC2 degradation (Fig. 3c).

To rule out the possibility that the decrease in FOXC2 protein levels, elicited by BI 2536 treatment, is due to arrest at prometaphase—when FOXC2 protein levels are normally low—we treated SUM159 cells with nocodazole (100 nM) or BI 2536 (50 nM). After treatment, we harvested the floating prometaphase cells and attached late G2 cells and analysed the FOXC2 protein levels. Following nocodazole treatment, attached late G2 cells had elevated FOXC2 levels whereas the floating cells—enriched for prometaphase—showed significantly lower FOXC2 levels, as expected (Fig. 3d). However, when cells were treated with BI 2536, FOXC2 levels were dramatically lower in both the attached late G2 and floating mitotic cell populations (Fig. 3d). In addition, we also performed immunofluorescence staining for FOXC2 and PLK1 in SUM159 cells following exposure to hydroxyurea to block cells in G1/S boundary and released cells from the block by removing it from the media to progress them to the G2 and M phases, by fixing them 4 and 8-hour post-release respectively. Interestingly, we observed co-staining of FOXC2 and PLK1 in the nucleus after 4-hour release following hydroxyurea arrest during G2, but we did not see the same phenomenon, due to decreased FOXC2 protein levels, during mitotic progression (Fig. 3e). These findings suggest that the activity of PLK1 is essential for FOXC2 stability at G2.

### FOXC2 regulates the G2/M transition of stem cell-enriched cells

**Figure 4:**
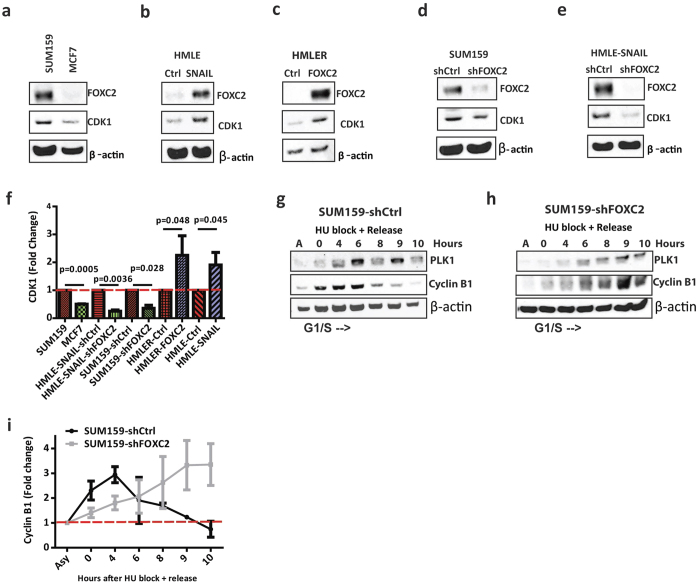
FOXC2 regulates mitotic entry of CSC-enriched TNBC cells and cells that have undergone EMT by enhancing the expression of CDK1, a master regulator of the G2/M transition. (**a**) Immunoblotting for FOXC2 and CDK1 in SUM159 and MCF7 cells. (**b**) Immunoblotting for FOXC2 and CDK1 in HMLE-Ctrl and HMLE-SNAIL cells. (**c**) Immunoblotting for FOXC2 and CDK1 in HMLER-Ctrl and HMLER-FOXC2 cells. (**d**) Immunoblotting for FOXC2 and CDK1 in SUM159-shCtrl and SUM159-shFOXC2 cells. (**e**) Immunoblotting for FOXC2 and CDK1 in HMLE-SNAIL-shCtrl and HMLE-SNAIL-shFOXC2 cells. (**f**) Quantification of CDK1 protein levels were performed from three independent replicates. CDK1 protein levels were normalized to β -actin. The statistical significance of the differences in CDK1 levels was determined by Student’s t-test. Data are presented as mean ± SD. (**g**,**h**) SUM159-shCtrl and SUM159-shFOXC2 cells were arrested with hydroxyurea and released to allow cell cycle progression and mitotic entry. PLK1 and cyclin B1 levels were detected by immunoblotting as indicators of the timing of mitosis in SUM159-shCtrl (**g**) and SUM159-shFOXC2 (**h**) cells. β-actin was used as a loading control. (**i**) The protein levels of cyclin B1 was quantified from the three independent replicates of SUM159-shCtrl and SUM159-shFOXC2 after hydroxyurea block and release. Cyclin B1 levels were normalized to β -actin. Data are presented as mean ± S.D.

The fact that FOXC2 protein level increases during G2, and dramatically decreases upon mitotic entry, suggests that FOXC2 can participate in the regulation of the G2/M transition. Consistent with a role for FOXC2 in the regulation of the cell cycle, mutation of eight FOXC2 phosphorylation sites resulted in the downregulation of genes encoding many mitotic proteins, including the master regulator of the G2/M transition CDK1^29^. To ascertain whether FOXC2 regulates the G2/M transition, we tested whether there is a link between FOXC2 and CDK1 expression in stem cell-enriched cells. Immunoblotting showed that FOXC2-expressing TNBC SUM159 cells exhibit higher CDK1 expression (p = 0.0005) compared to FOXC2-negative luminal A MCF7 cells (Fig. 4a,f). Moreover, HMLE-SNAIL cells, which express high levels of endogenous FOXC2 relative to control vector cells, also expressed more than two-fold CDK1 (Fig. 4b; p = 0.045). Similarly, HMLER-FOXC2 cells, which express FOXC2 from an exogenous vector relative to control vectors express more than two-fold CDK1 levels compared to control vector-transduced epithelial counterparts (Fig. 4b,c; p = 0.048). To further confirm the role of FOXC2 in the regulation of CDK1, we silenced FOXC2 in HMLE-SNAIL cells (p = 0.0036) as well as in SUM159 cells (p = 0.028) using shRNA, and observed a significant decrease in CDK1 expression compared to control shRNA-expressing cells (Fig. 4d,e). These findings suggest that FOXC2 may regulate the expression of CDK1 and, thus, could regulate the G2/M transition. To test the role of FOXC2 in the regulation of the G2/M transition, we arrested SUM159 cells expressing control shRNA (SUM159-shCtrl) and SUM159-shFOXC2 cells, at G1/S using hydroxyurea. After hydroxyurea treatment, we released the cells and harvested cell lysates for immunoblotting at 0, 4, 6, 8, 9 and 10-hour post-release. Phase-contrast images of hydroxyurea-synchronised cells show that SUM159-shCtrl cells are highly enriched in mitotic cells between 6-8 hour post-release, whereas SUM159-shFOXC2 cells only begin to enter mitosis after 9–10 hour (Supplementary Fig. 2a,b). To ascertain the timing of mitotic entry in more detail, we analysed the expression of PLK1 and cyclin B1, which accumulate at late G2 and mitosis respectively. Moreover, we quantified the levels of cyclin B1 at different time points from three independent replicates (Fig. 4i). SUM159-shCtrl cells show increased PLK1 and cyclin B1 levels 4–6 hour post-release (Fig. 4g,i), whereas in SUM159-shFOXC2 cells PLK1 and cyclin B1 levels accumulate 8–10 hour post-release (Fig. 4h,i). However, we did not see a significant difference in proliferation of HMLE-SNAIL-shFOXC2 and SUM159-shFOXC2 and HMLE-SNAIL-shCtrl and SUM159-shCtrl cells respectively, or between HMLER-FOXC2 and HMLER-vector Ctrl counterparts (Supplementary Fig. 2c–e).

### FOXC2 expressing CSC-enriched TNBC cells are sensitive to PLK1 inhibition

**Figure 5:**
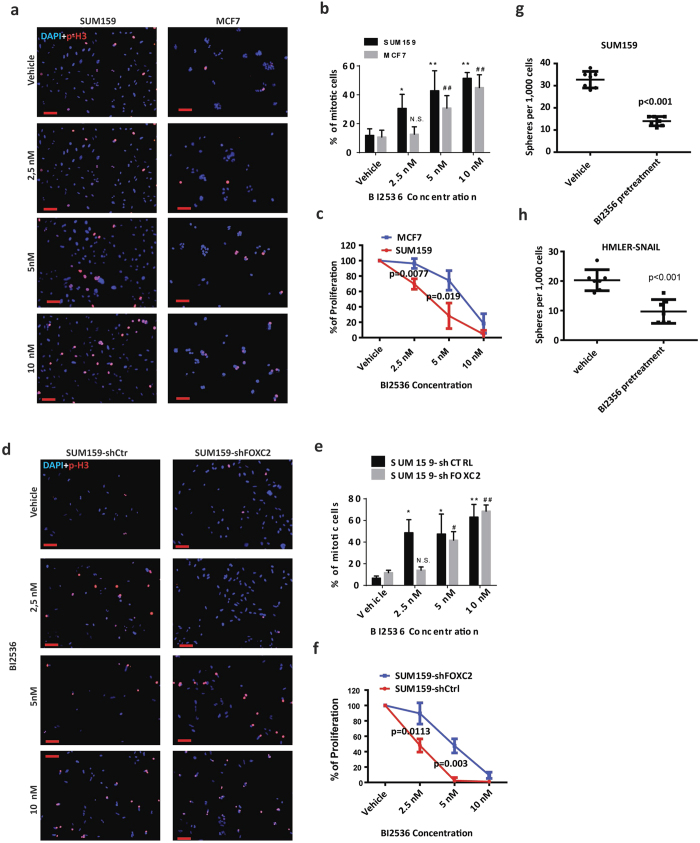
FOXC2 expression correlates with the sensitivity of CSC-enriched cells to the PLK1 inhibitor BI 2536. (**a**) Immunostaining of SUM159 and MCF7 cells, treated with BI 2536 for 24 h, for p-H3 (red). Nuclei were counterstained with DAPI (blue) Scale bar 100 µm. (**b**) Quantification of p-H3 positive cells following treatment of SUM159 and MCF7 cells with increasing concentrations of BI 2536 for 24 h. * and ** indicate Student’s t-test p values of p < 0.05 and p < 0.01 respectively, when counts are compared between vehicle- and BI 2536-treated SUM159 cells. ^##^indicates Student’s t-test p value of p < 0.01 when counts are compared between vehicle- and BI 2536-treated MCF7 cells. (**c**) The viability of SUM159 and MCF7 cells, treated with the indicated concentrations of BI 2536 for 48 h, was determined. Statistical significances were calculated using Student’s t-test. (**d**) Immunofluorescence staining for p-H3 (red) in SUM159-shCtrl and SUM159-shFOXC2 cells treated with increasing concentrations of BI 2536 for 24 h. Nuclei were counterstained with DAPI (blue) Scale bar 100 µm. (**e**) Quantification of p-H3 positive cells after treatment of SUM159-shCtrl and SUM159-shFOXC2 cells with increasing concentrations of BI 2536 for 24 h. * and ** indicate Student’s t-test p value of p < 0.05 and p < 0.01 respectively when compared between vehicle- and BI 2536-treated SUM159-shCtrl cells. ^#^and ^##^indicate Student’s t-test p value of p < 0.05 and p < 0.01 respectively when compared between vehicle- and BI 2536-treated SUM159-shFOXC2 cells. (**f**) SUM159-shCtrl and SUM159-shFOXC2 cells were treated with increasing concentrations of BI 2536 for 48 h and the percentage of proliferation was determined. Statistical significances were calculated using Student’s t-test. (**g**) A mammosphere formation assay was performed using SUM159 cells pretreated with 80 nM BI 2536 for 24 h. The statistical significance of the differences in mammosphere-forming efficiency, between vehicle- and BI 2536-pretreated SUM159 cells, was determined by Student’s t-test (**p < 0.01). (**h**) A mammosphere formation assay was performed using HMLER-SNAIL cells, pretreated with 80 nM BI 2536 for 24 h. The statistical significance of the differences in mammosphere-forming efficiency, between vehicle- and BI 2536-pretreated SUM159 cells, was determined by Student’s t-test (**p < 0.01). Data are presented as mean ± SD.

Previously, it was shown that CSC-enriched TNBC cells are sensitive to G2/M kinase inhibitors, such as the PLK1 inhibitor BI 2536^21^,^22^. Here, we have shown that FOXC2 regulates the G2/M transition of CSC-enriched cells and boosts their mitotic entry. Accordingly, we tested whether FOXC2 expression could confer sensitivity of CSC-enriched cells to PLK1 inhibitor. Since inhibition of PLK1 has been reported to cause mitotic arrest with subsequent induction of cell death^38^, we treated FOXC2-expressing SUM159 and FOXC2-negative MCF7 cells with different concentrations of BI 2536, and performed p-H3 immunofluorescent staining to determine the percentage of mitotically arrested cells after 24 h of treatment (Fig. 5a). To begin, we found that SUM159 cells undergo mitotic arrest at lower BI 2536 concentrations (2.5 nM) compared to MCF7 cells (5.0 nM), indicating that the FOXC2 expressing cells show higher sensitivity to PLK1 inhibitor (Fig. 5b). Accordingly, SUM159 cells exhibited a decrease in proliferation by about 30% at 2.5 nM BI 2536 and by 80% at 5 nM BI 2536, when compared to vehicle-treated counterparts. These figures contrast sharply with MCF7 cells that showed only a 5% reduction in proliferation at 2.5 nM BI 2536 (p = 0.0077 when compared to SUM159) and 20% decreased proliferation at 5 nM BI 2536 (p = 0.019 when compared to SUM159), suggesting that MCF7 cells are more resistant to PLK1 inhibition (Fig. 5c). However, at 10 nM BI 2536, the proliferation was inhibited equally in both cell lines. These results indicate that TNBC FOXC2-expressing cells show higher sensitivity to BI 2536 compared to FOXC2-negative luminal MCF7 cells. To further test if FOXC2 expression correlates with sensitivity to BI 2536, we used SUM159-shCtrl and SUM159-shFOXC2 cells. SUM159-shCtrl cells showed a significant increase from 6.7 ± 1.9% to 48.6 ± 12.1% (p = 0.034) in p-H3 positive cells, after 24 h of treatment with 2.5 nM BI 2536 (Fig. 5d,e). Interestingly, in SUM159-shFOXC2 cells, treated for 24 h with 2.5 nM BI 2536, there was no statistically significant increase in p-H3 positive cells (from 11.7 ± 2.2% to 14.1 ± 2.9%). It was noted that with increasing BI 2536 concentrations, the proliferation of SUM159-shCtrl cells was significantly lower compared to SUM159-shFOXC2 cells (at 2.5 nM BI 2536 p = 0.0113 and at 5 nM BI 2536 p = 0.003). Again, at 10 nM BI 2536, the proliferation was inhibited in both cell lines.

Since FOXC2 has strongly been linked to CSC properties, such as self-renewal, which is measured *in vitro* by sphere formation assays, we tested if intact PLK1-FOXC2 signalling is essential for sphere formation. We pre-treated SUM159 and HMLER-SNAIL cells with 80 nM BI 2536 for 24 h, sufficient to elicit a decrease in FOXC2 levels. Interestingly, transient BI 2536 pre-treatment inhibited the sphere-forming efficiency of SUM159 and HMLER-SNAIL cells, without compromising their long-term proliferative capacity in 2D culture (Fig. 5g,h; Supplementary Fig. 3). While our data demonstrate that FOXC2 expression correlates with the sensitivity of CSC-enriched cells to PLK1 inhibition and that the PLK1-FOXC2 signalling link might be essential for maintaining self-renewal capabilities *in vitro,* additional experiments are necessary to prove this in detail. To investigate the clinical significance of the upstream (PLK1) and downstream (CDK1) modulators of FOXC2, we analyzed the data from a cohort of patients with invasive breast cancers following taxane-anthracycline chemotherapy^39^. Interestingly, whereas PLK1 alone did not have predictive power (p = 0.052), high CDK1 expression predicted improved Distant Relapse-Free Survival (DRFS) compared to low CDK1 (p = 0.0048) (Supplementary Fig. 4a,b). However, when combined high CDK1 and PLK1 expression levels compared to combined low CDK1 and PLK1, the high levels of CDK1 and PLK1 predicted significantly poorer DRFS than the combined low levels of CDK1 and PLK1 (p = 0.0042) (Supplementary Fig. 4c).

## Discussion

Recent studies have shown that the CSC-rich CD44^high^ subpopulations of head and neck, breast and prostate carcinoma cells express higher levels of G2/M checkpoint proteins compared to CD44^low^ counterparts^40^. Indeed, CSCs have also been shown to be exquisitely sensitive to inhibitors of G2/M transition regulators, such as PLK1 and Aurora A, compared to the more differentiated cells of the tumour bulk, which in contrast are more sensitive to anti-mitotic drugs^4^,^16^,^17^,^18^,^19^,^20^,^21^,^22^,^41^,^42^. Accordingly, CSCs and the differentiated cells exhibit differences in the regulation of the cell cycle and the response to DNA damage, which are controlled by many G2/M kinases. PLK1 is involved in several aspects of cell cycle regulation including the G2/M transition and mitosis^16^,^17^. Most recently, PLK1 has been identified as a potential therapeutic target in TNBC^22^. While the CSCs are believed to be slow cycling, the molecular mechanism by which PLK1 sensitizes CSC-rich TNBCs remains unknown.

FOXC2 was shown to be phosphorylated at multiple sites in a cell cycle-dependent manner, with the highest levels of phosphorylation detected during G2/M^43^. Moreover, Ivanov *et al.*^29^ have shown that, in lymphatic endothelial cells, FOXC2 activity is regulated by phosphorylation and that mutating key phosphorylation sites in FOXC2 inhibit chromatin binding, causing differences in the gene expression profile of many mitotic proteins or G2/M regulators. These findings suggest that FOXC2 may regulate the cell cycle in CSC-rich cell populations. In fact, in this study, we found that FOXC2 expression is regulated in a cell cycle-dependent manner with an accumulation of FOXC2 protein levels at G2, followed by rapid degradation upon mitotic entry. Moreover, FOXC2 is also an important downstream player of PLK1 in cancer cells with stem cell properties.

While many Forkhead transcription factors have been implicated in the regulation of the cell cycle^44^, the fact that FOXC2 expression is restricted to cancer cells with stem cell properties, as well as its central role in the regulation of EMT and metastasis, attest to its potential utility as a therapeutic target^28^. Most interestingly, we noticed that PLK1 activity is necessary for FOXC2 protein stability suggesting that the fluctuation of FOXC2 protein levels, during the cell cycle, may be regulated by PLK1. Several cell cycle-related proteins are known to be phosphorylated by PLK1, thus impacting their stability and/or activity, and ensuring timely mitotic entry and progression^45^,^46^,^47^,^48^. Even though the detailed mechanism of FOXC2 regulation by PLK1 remains unclear and also we cannot rule out the possible off-target effect of BI 2536 either, our finding that inhibition of PLK1 by BI 2536 caused a decrease in FOXC2 protein levels reiterates that PLK1 regulates FOXC2 expression. For example, PLK1-dependent phosphorylation of FOXM1 has been shown to prevent its sumoylation and proteolytic degradation and facilitate FOXM1 mitotic functions^49^,^50^. Similarly, we hypothesize that PLK1 activity may antagonize or promote other post-translational modifications of FOXC2 and, thus, maintain its stability^51^. The fact that PLK1 activity is required to maintain FOXC2 protein stability is interesting since PLK1 inhibition has also been shown to inhibit CSC properties, such as sphere formation and tumour growth in xenograft models, through inhibition of CSC-specific factors^21^,^22^,^52^. In line with this, we also found that PLK1 activity is necessary to maintain FOXC2 stability/activity in CSC-enriched cells and thus helps support self-renewal properties, such as sphere formation.

The proper regulation of the G2/M transition is likely to be important for CSCs for several different reasons. Firstly, CSCs are slow-cycling and have a high capacity for DNA repair, compared to differentiated cells, owing to the activity of several G2/M checkpoint kinases that instigate an efficient DNA damage response that contributes to therapy resistance^4^,^38^,^53^,^54^,^55^. Secondly, CSCs have the unique ability to divide either asymmetrically or symmetrically and, therefore, the control of mitosis will influence the proportion of CSCs within a tumour^56^,^57^,^58^. Although FOXC2 was previously shown to promote the proliferation of several cancer cell types^30^,^31^,^32^,^33^,^34^,^35^, we show here, for the first time, that FOXC2 regulates the G2/M transition of CSC-enriched TNBC cells and immortalized cells that have undergone EMT. Mechanistically how FOXC2 enhances mitotic entry remains unclear, but we found a tight correlation between FOXC2 and mitotic kinase CDK1 expression. In fact, a link between FOXC2 activation and CDK1 expression was shown previously^29^. Moreover, the significance of this observation is reiterated by our finding that PLK1 and the FOXC2 potential target CDK1 mRNA collectively predict poor DRFS among invasive breast cancer patients after taxane-anthracycline chemotherapy^39^. Even though overall CDK1 expression levels^59^,^60^ predicted better DRFS, similarly, PLK1 alone in cancers, including breast cancer, has been shown to be weak and inconsistent^61^,^62^,^63^, in combination both CDK1 and PLK1 predicted poor survival. FOXC2 mRNA levels did not predict clinical outcome, either alone or in combination with PLK1 or CDK1, in line with our previous findings that only FOXC2 protein levels^27^, and not its transcript levels^64^, are capable of predicting clinical outcome.

Although anti-mitotic agents are known to kill the differentiated cells of the tumour bulk, eliciting tumour shrinkage, they leave CSCs unscathed leading to tumour relapse and CSC enrichment^3^,^4^,^5^,^6^. Paradoxically several recent papers have shown that CSCs are more sensitive to G2/M kinase inhibitors, such as those targeting PLK1 and Aurora A^19^,^20^,^21^,^22^,^39^,^40^. Our findings that targeting PLK1 reduces FOXC2 provides a molecular clue, how G2/M kinases, in this regard PLK1, may sensitize CSCs to mitotic drugs or cell cycle inhibitors. Accordingly, this study suggests that inhibiting PLK1-FOXC2 signalling could be useful in inhibiting CSC-rich TNBCs.

## Methods

### Cell culture

HMLE-Ctrl, HMLE-SNAIL, HMLE-SNAIL-shCtrl and HMLE-SNAIL-shFOXC2 cells were generated previously and maintained as described^28^. SUM159 cells were cultured as described previously^28^. SUM159-shCtrl and SUM159-shFOXC2 cells were generated and maintained as described previously^28^. Hs578T cells were grown in DMEM/F12 supplemented with 10% foetal bovine serum and penicillin/streptomycin. Ras-transformed HMLE cells (HMLER) overexpressing FOXC2 (HMLER-FOXC2), SNAIL (HMLER-SNAIL) or control vector (HMLER-Ctrl) were generated previously and maintained as described^28^.

### Inhibitors and drugs

The following drugs and inhibitors were used: BI 2536 (Selleckhem, Houston, TX, USA), CDK1 inhibitor (EMD Millipore, Temecula, CA, USA), nocodazole (Sigma-Aldrich, St Louis, MO, USA), hydroxyurea (Sigma-Aldrich) and MG132 (Sigma-Aldrich). All drugs were reconstituted in DMSO and DMSO alone was used as vehicle.

### Cell synchronisation and cell cycle analysis

G2/M arrest was achieved by synchronising cells with 100 nM nocodazole. After 14 h, the cells were washed three times with PBS and released into fresh warm medium without nocodazole. G1/S arrest was achieved by treating cells with 2 mM hydroxyurea. After 14 h, the cells were washed and released into fresh medium without hydroxyurea. Cells were washed with ice-cold PBS and harvested by trypsinization. Cell pellets were resuspended in ice-cold PBS, fixed in ice-cold 70% ethanol and stained with 20 μg/ml propidium iodide (PI; Sigma-Aldrich), with RNAase A (Qiagen, Germantown, Maryland, USA), prior to cell cycle analysis by BD Accuri C6. The total number of analysed cells was 10, 000. FlowJo was used for cell cycle analysis (FlowJo, LLC, Ashland, OR, USA).

### Immunoblotting

For immunoblotting, proteins were extracted by lysing cells in ice-cold radioimmunoprecipitation buffer supplemented with protease and phosphatase inhibitors (Roche, Nutley, NJ, USA). Protein was quantified using the Bradford assay (Bio-Rad, Hercules, CA, USA) and 30 μg of total protein was resolved by SDS-PAGE and transferred to polyvinylidene fluoride membranes. Membranes were blocked with 5% skim milk in TBST (10 mM Tris, pH 8.0, 150 mM NaCl, 0.5% Tween 20) for 1 h, washed with TBST and incubated with antibodies against: PLK1 (Cell Signalling, Danvers, MA, USA), CDK1 (Abcam, Cambridge, UK), cyclin B1 (Abcam), phospho-Histone H3 (phospho-Ser10, Abcam), β-actin (Sigma-Aldrich) and FOXC2 (kindly provided by Dr. Naoyuki Miura, Hamamatsu University School of Medicine, Hamamatsu, Japan). For quantifications, the levels of protein was measured by ImageJ and band intensities were normalized to corresponding loading controls.

### Proliferation assays

Cells were seeded at a density of 50,000 cells per well of 24-well plates. One day after plating, the culture medium was replaced with medium containing the indicated concentrations of BI 2536 or vehicle. After 48 h, cells were trypsinized and counted with a haemocytometer. Cell viability was determined by trypan blue exclusion. The percentage of proliferation was calculated as the percentage of viable cells in BI 2536-treated cells, relative to vehicle-treated counterparts, at each dose. Each experiment was performed three times with triplicates samples.

### Immunofluorescence

Cells were plated on pre-sterilised cover slips. Cells were fixed and permeabilized with 4% paraformaldehyde +0.1% Triton-X 100 for 20 min at +4^o^ C. Paraformaldehyde was quenched by 5% glycine for 15 min at room temperature. Samples were blocked by 4% bovine serum albumin (BSA) in phosphate-buffered saline (PBS) for 1 h at room temperature. Staining was performed using the aforementioned primary antibodies along with anti-alpha tubulin (Cell Signalling)) diluted in 4% BSA in PBS. Species-specific Alexa Fluor 488- and Alexa Fluor 568-conjugated secondary antibodies (Life Technologies, Grand Island, NY, USA) were diluted 1:1000 in 4% BSA in PBS and applied for 1 h at room temperature. Nuclei were counterstained with 4′,6-diamidino-2-phenylindole (DAPI; Molecular Probes, Grand Island, NY, USA). The coverslips were mounted onto glass slides with DAKO fluorescent mounting medium (DAKO, Carpinteria, CA, USA).

### Quantification of p-H3 positive cells

Cells were plated on pre-sterilized cover slips (50 000 cells per well in 24-well plate) a day before the treatment was started. Cells were treated with the indicated concentrations of BI 2536 for 24 h, fixed and immunostained for p-H3 as described above. The number of p-H3 positive cells and the total cell number, as indicated by the number of DAPI-stained nuclei, were counted in 10 randomly selected microscope fields per sample (imaged with a 10x objective). The percentage of p-H3 positive cells was then calculated. The experiment was repeated twice with each cell line.

### Mammosphere assays

Mammosphere assays were performed as previously described^28^. Briefly, cells were seeded at a density of 1,000 cells per well in ultralow attachment 96-well plates (Corning, Glendale, AZ, USA) and cultured for 7–10 days. Medium was replenished every three days. Mammospheres with a diameter larger than 70 micrometers were counted.

### Statistical analyses

Unless otherwise stated, each experiment was repeated at least 3 times or more, with at least 3 technical replicates. Data are presented as mean ± SD. Student’s t-test (unpaired, two-tailed) was used to compare 2 groups of independent samples.

### Statistical analysis of predictive performance of PLK1 and/or CDK1 in patients with invasive breast cancers after taxane-anthracycline chemotherapy

The patient datasets analysed were previously described^34^. All gene expression microarrays of diagnosed breast cancer were profiled (U133A Gene Chip; Affymetrix, Santa Clara, California) in the Translational Molecular Pathology Department at the MD Anderson Cancer Centre, Houston, Texas. Distant relapse–free survival (DRFS) was defined as the interval from initial diagnostic biopsy until diagnosis of distant metastasis or death from breast cancer, non-breast cancer, or unknown causes^60^. Statistical computation of Kaplan-Meier estimates of DRFS were performed in R version 2.10.1 and Bioconductor version 3.1.

## Additional Information

**How to cite this article**: Pietilä, M. *et al.* FOXC2 regulates the G2/M transition of stem cell-rich breast cancer cells and sensitizes them to PLK1 inhibition. *Sci. Rep.* **6**, 23070; doi: 10.1038/srep23070 (2016).

## Supplementary Material

Supplementary Information
