# Supplementary material for: FOXC2 regulates the G2/M transition of stem cell-rich breast cancer cells and sensitizes them to PLK1 inhibition
Source: Sci Rep. 2016 Apr 11;6:23070. doi: 10.1038/srep23070 (PMC4827390; doi:10.1038/srep23070)
Supplement: Supplementary Information [file srep23070-s1.pdf]

FOXC2 regulates the G2/M transition of stem cell-rich breast cancer cells and sensitizes them to PLK1 inhibition

Mika Pietilä[1], Geraldine V. Vijay[1], Rama Soundararajan[1], Xian Yu[1], William F. Symmans[1], Nathalie Sphyris[1] & Sendurai A. Mani[1,2,3,] *

[1]Department of Translational Molecular Pathology, The University of Texas MD Anderson Cancer Centre, Houston, TX, USA

[2]Metastasis Research Centre, The University of Texas MD Anderson Cancer Centre, Houston, TX, USA

[3]Center for Stem Cells and Developmental Biology, The University of Texas MD Anderson Cancer Centre, Houston, TX, USA

*****Correspondence and requests for materials should be addressed to S.A.M. (e-mail: smani@mdanderson.org)

Contents:

Supplementary Figure 1. PLK1 and not CDK1 or Aurora A kinase sites are evolutionary conserved

Supplementary Figure 2. FOXC2 expression regulates mitotic entry of CSC-enriched TNBCs without significant impact on overall proliferation rate.

Supplementary Figure 3. BI 2536 pretreatment does not induce an irreversible proliferation arrest in 2D monolayer cultures of SUM159 and HMLER-SNAIL cells.

Supplementary Figure 4. Clinical predictive significance of the expression of PLK1 and CDK1 alone, or in combination, in invasive breast cancer patients after taxane-anthracycline chemotherapy.

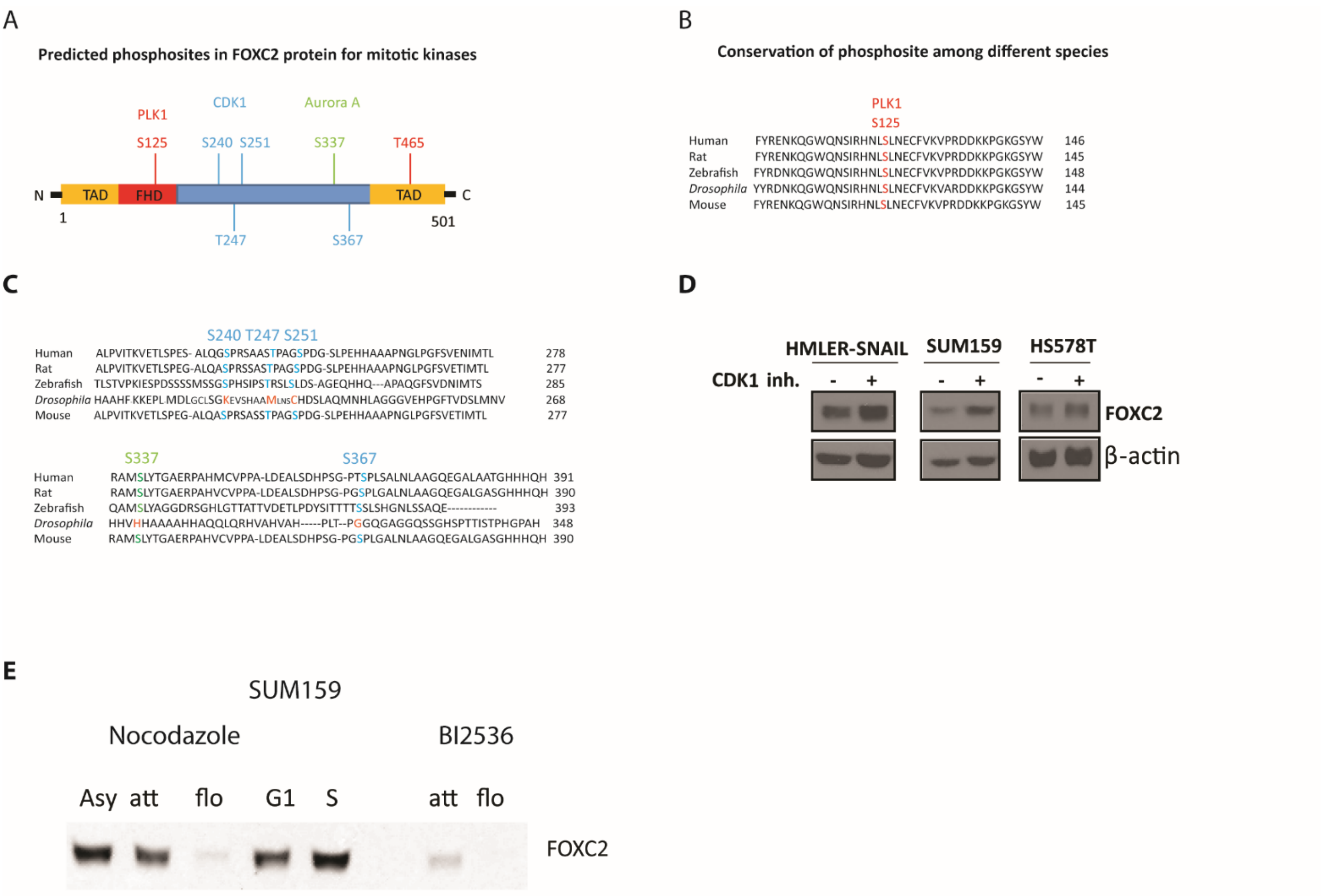

Supplementary Figure 1. PLK1 and not CDK1 or Aurora A kinase sites are evolutionary conserved. (**a**) Schematic representation of the FOXC2 protein showing predicted phosphorylation sites for only mitotic kinases are shown. Numbers represent aminoacid positions. TAD, transactivation domain; FHD, forkhead DNA binding domain. (**b**) BLAST alignment of UniProt FOXC2 aminoacid sequences from human, rat, zebrafish, *Drosophila* and mouse shows high evolutionary sequence conservation at serine 125 (S125), the putative phosphorylation site for PLK1. **(c)** BLAST alignment of UniProt FOXC2 aminoacid sequences from human, rat, zebrafish, *Drosophila* and mouse shows lack of evolutionary sequence conservation at the predicted CDK1 and Aurora A phosphosites in FOXC2. (**d**) Inhibition of CDK1 did not induce degradation of FOXC2, as determined by immunoblotting. β-actin was used as a loading control. (**e**) Original blot images of data, depicted in Fig. 3f, indicating that samples were analysed on the same gel and are, thus, directly comparable.

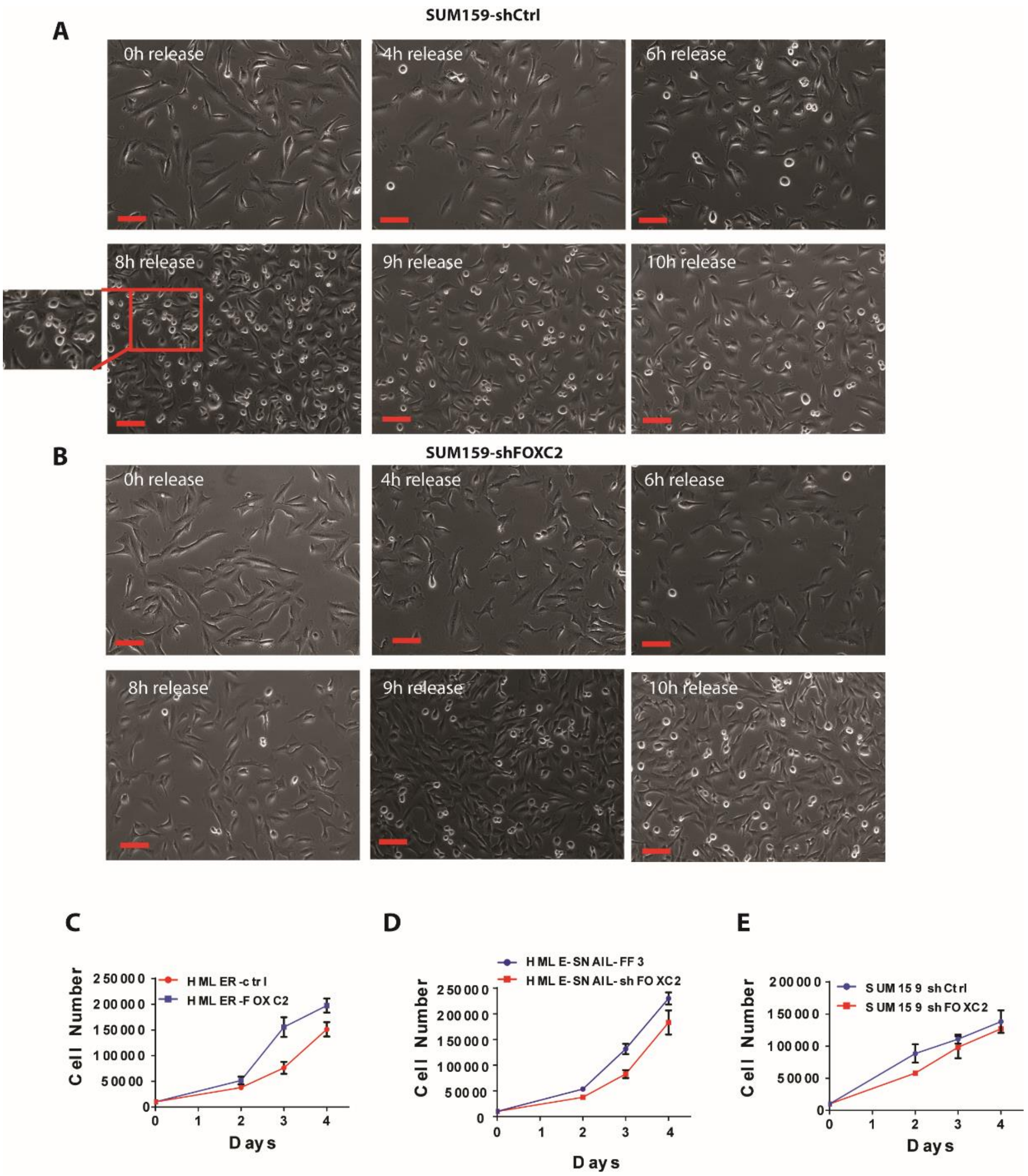

Supplementary Figure 2. FOXC2 expression regulates mitotic entry of CSC-enriched TNBCs without significant impact on overall proliferation rate. (**a, b**) Phase contrast images of SUM159-shCtrl (a) and SUM159-shFOXC2 (b) cells after hydroxyurea treatment and release. Magnified insert shows the characteristic morphology of rounded, semi-detached mitotic cells (10x objective). (**c-e**) Proliferation assays were performed by obtaining viable cell counts at indicated timepoints of HMLER-Ctrl and HMLER-FOXC2 (c), HMLE-SNAIL-shCtrl and HMLE-SNAIL-shFOXC2 (d), and SUM159-shCtrl and SUM159-shFOXC2 cells (e).

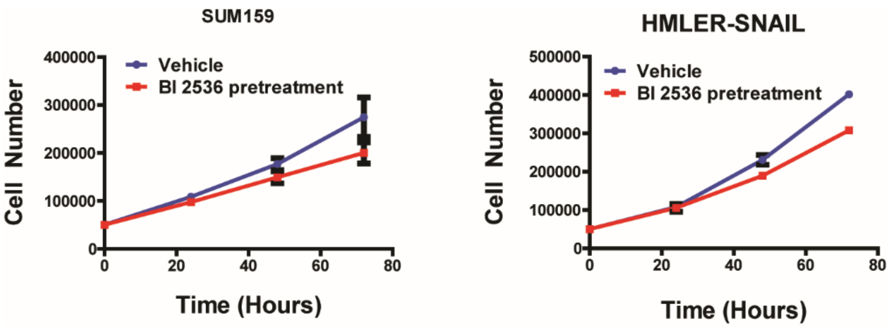

Supplementary Figure 3. BI 2536 pre-treatment does not induce an irreversible proliferation arrest in 2D monolayer cultures of SUM159 and HMLER-SNAIL cells. The proliferation of SUM159 and HMLER-SNAIL cells, pre-treated with 80 nM BI 2536 or vehicle for 24 h, was measured. Pre-treated cells were subsequently trypsinised and subjected to a mammosphere assay, as presented in Fig. 5g,h.

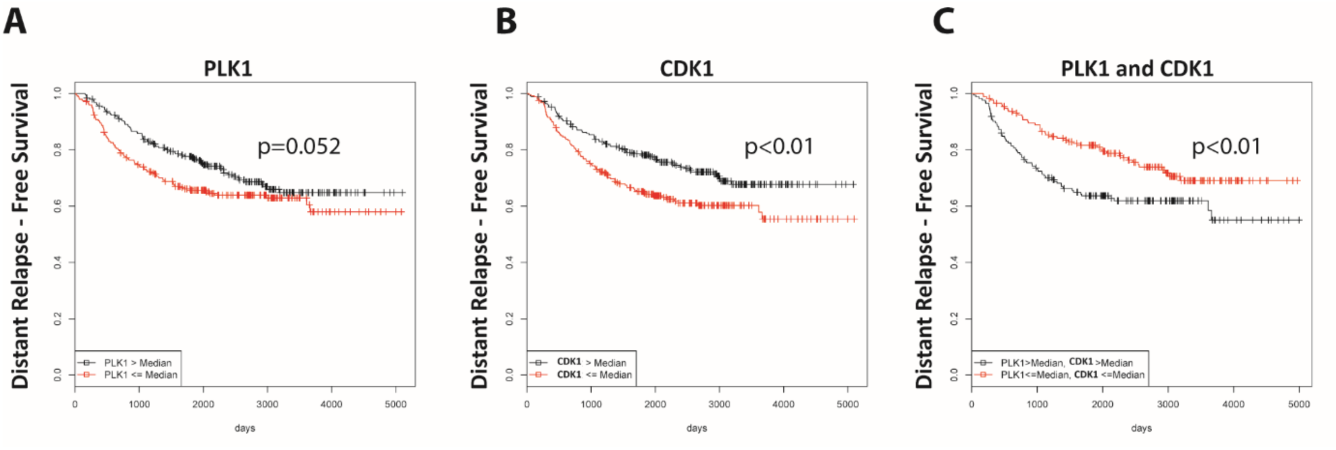

Supplementary Figure 4. Clinical predictive significance of the expression of PLK1 and CDK1 alone, or in combination, in invasive breast cancer patients after taxane-anthracycline chemotherapy. Tumour biopsy samples were obtained from patients with invasive breast cancer prior to and following taxane-anthracycline chemotherapy. Gene expression microarrays of tumours were profiled using the U133A Gene Chip (Affymetrix, Santa Clara, California, USA). The Distant Relapse–Free Survival of PLK1 and CDK1 gene expression were dichotomized at their median and compared using Kaplan-Meier analysis. (**a**) Kaplan-Meier estimates of distant relapse – free survival based on PLK1 expression. Black line indicates PLK1 levels above the median and red line PLK1 levels equal or below the median. (**b**) Kaplan-Meier estimates of distant relapse – free survival based on CDK1 expression. Black line indicates CDK1 levels above the median and red line CDK1 levels equal or below the median. (**c**) Kaplan-Meier estimates of distant relapse – free survival based on combined CDK1 and PLK1 expression. Black line indicates combined PLK1 and CDK1 levels above the median and red line combined PLK1 and CDK1 levels equal or below the median.
